# Outcome of parasitological examinations in dogs in Germany: a retrospective survey

**DOI:** 10.1007/s00436-024-08181-6

**Published:** 2024-03-14

**Authors:** Jacqueline Csokai, Anton Heusinger, Elisabeth Müller

**Affiliations:** 1 Laboklin GmbH & Co. KG, Paul-Hahn-Straße 3, 4020 Linz, Austria; 2 Laboklin GmbH & Co. KG, Steubenstraße 4, 97688 Bad Kissingen, Germany

**Keywords:** Dogs, Endoparasites, Prevalence, Germany, ELISA, PCR

## Abstract

Dog faecal samples examined from January 2019 to December 2019 were retrospectively analysed for frequency of endoparasites. The examinations were performed with several different methods: 29,219 samples were examined by flotation method and sodium acetate-acetic acid-formalin concentration (SAFC) technique, 1,330 samples by Baermann-Wetzel migration technique, 12,221 samples using a *Giardia* coproantigen enzyme-linked-immunosorbent assay (ELISA), 1,180 samples using a *Cryptosporidium* coproantigen ELISA, 1,671 samples by polymerase chain reaction (PCR) testing for *Giardia duodenalis* and 447 samples by PCR testing for *Cryptosporidium* spp.. A total of 7.1% of the samples were positive for parasites in the microscopical examination using the flotation method and SAFC technique. The parasites found included *Cystoisospora* spp. (2.8%), *Giardia duodenalis* (2.3%), Ancylostomatidae (1.8%), *Toxocara canis* (1.6%), *Trichuris vulpis* (0.7%), *Toxascaris leonina* (0.5%), *Capillaria* spp. (0.2%), *Angiostrongylus vasorum* (0.2%), *Crenosoma vulpis* (0.1%), Taeniidae (0.1%), *Sarcocystis* spp. (0.03%), *Dipylidium caninum* (0.01%), *Diphyllobothrium latum* (< 0.01%), Spirurida (< 0.01%) and Opisthorchiidae (< 0.01%). Using the Baermann-Wetzel migration technique, *Angiostrongylus vasorum* was found in 0.75% and *Crenosoma vulpis* in 0.3% of the samples. ELISAs for *Giardia duodenalis* and *Cryptosporidium* spp. revealed 13.9% and 1.0% positive faecal samples, and *Giardia duodenalis* and *Cryptosporidium* spp. PCRs 19.4% and 2.0%, respectively. Dogs in the first year of life were more frequently infected with parasites than older animals. In the microscopic examination using flotation method and SAFC technique, the significantly highest detection rates were found in dogs up to six months of age (*p* < 0.001).

## Introduction

Dogs and cats are popular pets in Germany. While the level of veterinary care for these animals is generally high, endoparasites remain a consideration in patients with diarrhoea. Especially in puppies (Duijvestijn et al. 2016; Grellet et al. 2014) endoparasites are an important differential diagnosis due to increased infection pressure during breeding, specific transmission routes such as intrauterine and lactogenic transmission of *Toxocara canis* and lactogenic transmission of *Ancylostoma caninum*, as well as the immature immune system of young animals which contribute to increased infection rates and greater difficulty in controlling parasites (Burke and Roberson 1985; Pereira et al. 2019). Some parasites are also important as zoonoses. Toxocariasis in humans is caused by larvae of *Toxocara canis* and can occur as visceral larva migrans, ocular larva migrans or neurotoxocariasis. The infection is often asymptomatic or rarely part of the differential diagnosis on examination, making it difficult to estimate the true incidence. The seroprevalence in German-speaking countries is between 1.4% and 6.3% (Strube el al. 2020). The reference centre in Austria records around 75 human cases per year (Auer and Aspöck 2014). Cutaneous larva migrans due to hookworm infection occurs rarely in Germany (Müller-Stöver et al. 2010). Echinococcosis is a very dangerous disease in humans. In 2020, 135 cases were reported to the Robert Koch Institute in Germany, 55 of which were infected in Germany (Robert Koch-Institut 2021). *Giardia duodenalis* consists of different genotypes and subgenotypes, with some subgenotypes of genotype A and B being considered zoonotic, while others are considered more host-specific (Feng and Xiao 2011).

Studies about the parasite infection rate help to raise the awareness of breeders, pet owners and veterinarians. They give an overview of which parasites occur most frequently. It is important to note that although retrospective studies of diagnostic laboratory results often include a large number of examined samples, they do not necessarily reflect true infection rates. In these studies, the investigated dog population is generally biased toward animals with gastrointestinal diseases as well as healthy dogs from owners with a higher-than-average commitment to their pet’s health, as they show interest in knowing if the animals have parasites or not by submitting samples (Barutzki and Schaper 2003, 2011; Elze et al. 2014; Gates and Nolan 2009; Raue et al. 2017). In addition, coproscopical examinations do not always detect a current infection, e.g. the low sensitivity of microscopic detection of tapeworm eggs due to the excretion of proglottids (Martínez-Carrasco et al. 2007).

The aim of this study was to provide an updated overview on the frequency of endoparasites detected in faecal samples from dogs submitted to a commercial veterinary laboratory.

## Materials and methods

Faecal samples from dogs submitted to a commercial veterinary laboratory (LABOKLIN GmbH & CO.KG, Bad Kissingen, Germany) were included in this retrospective study. Results of 34,624 samples, examined in 2019 (January until December), were evaluated. Samples were submitted by veterinarians or pet owners from all parts of Germany (Bavaria *n* = 9,573; North Rhine-Westphalia *n* = 6,031; Hesse *n* = 3,976; Lower Saxony *n* = 3,843; Baden-Württemberg *n* = 3,153; Rhineland-Palatinate *n* = 2,978; Schleswig–Holstein *n* = 1,153; Berlin *n* = 703; Brandenburg *n* = 661; Thuringia *n* = 555; Saarland *n* = 530; Saxony-Anhalt *n* = 412; Mecklenburg-Western Pomerania *n* = 409; Hamburg *n* = 280; Saxony *n* = 224; Bremen *n* = 143) for parasitological examination because of gastrointestinal diseases, therapy controls and routine examination of healthy animals. The different tests were requested by the customers as a single service or were included in diagnostic panels. Faecal samples were examined with one (*n* = 24,627) or more tests (*n* = 9,997), depending on the customer's request. Analysed tests (Table 1) for parasitological examinations were microscopic examination with the flotation method and the sodium acetate-acetic acid-formalin concentration (SAFC) technique (*n* = 29,219), microscopical examination with the Baermann-Wetzel migration technique (*n* = 1,330), coproantigen enzyme-linked-immunosorbent assay (ELISA) for *Giardia duodenalis* (*n* = 12,221) and *Cryptosporidium* spp. (*n* = 1,180), and polymerase chain reaction (PCR) for *Giardia duodenalis* (*n* = 1,671) and *Cryptosporidium* spp. (*n* = 447). Inclusion criteria for the retrospective analysis was provision of the dog`s age in the history. Only for the Baermann-Wetzel migration technique animals with and without information on age were included in the analysis due to a low number of positive samples.

**Table 1:** Number of faecal samples per test performed and age distribution of the samples analysed

Age groups	Number of examined samples (n) per test			
	Flotation method and SAFC technique	Baermann-Wetzel migration technique	*Giardia duodenalis* (ELISA and PCR)	*Cryptosporidium* spp. (ELISA and PCR)
≤ 3 months	1,134	N/A	692	70
> 3 – 6 months	1,489	N/A	1,109	97
≥ 6 – 12 months	2,344	N/A	1,568	155
≥ 1 – 5 years	10,759	N/A	5,144	572
≥ 5 – 10 years	8,119	N/A	3,143	404
≥ 10 years	5,374	N/A	2,236	329
Total	29,219	1,330	13,892 (ELISA: 12,221; PCR: 1,671)	1,627 (ELISA: 1,180; PCR: 447)

*Abbreviations*: *SAFC* sodium acetate-acetic acid-formalin concentration, *N/A* not available

The flotation method was performed with salt-glucose solution (specific gravity 1.3). The SAFC technique was conducted with the Mini Parasep® SF faecal parasite concentrator (Apacor Ltd., Wokingham, United Kingdom). The evaluation of egg/oocyst/cyst excretion in faecal samples was assessed semi-quantitatively and divided into low (1–10 counted eggs/oocysts/cysts per cover slip), moderate (11–25 counted eggs/oocysts/cysts per cover slip), high amount (> 25 counted eggs/oocysts/cysts per cover slip). Fifty-eight samples were excluded from the study because they contained eggs of parasites of other animals that do not parasitise dogs such as *Ascaridia* spp., *Moniezia* spp., oxyurids, *Fasciola hepatica*, or because only rarely isolated infections (*Dicrocoelium dendriticum*) were described, so that the detection in dog faeces is rather coincidental due to coprophagy or feeding of liver.

The examination for lungworm larvae using the Baermann-Wetzel migration technique (Deplazes et al. 2013) was not included in any diagnostic panel and therefore had to be ordered separately by the customers.

*Giardia* coproantigen ELISA was performed with RIDASCREEN® *Giardia* (R-Biopharm AG, Darmstadt, Germany) and *Cryptosporidium* coproantigen ELISA with RIDASCREEN® *Cryptosporidium* (R-Biopharm AG, Darmstadt, Germany) according to the manufacturer`s instructions. Real-time PCR was conducted for detection of *Giardia duodenalis* (Verweij et al. 2003) and conventional gel PCR for *Cryptosporidium* spp. (Richter et al. 2011).

Dogs whose faeces were microscopically examined for parasites by flotation method and SAFC technique were divided into six age groups to assess the correlation between age and infection rate (*n* = 29,219; Table 1). Parasites that were detected in less than 50 samples were excluded from the analysis (Taeniidae, *Sarcocystis* spp., *Diphyllobothrium latum*, *Dipylidium caninum*, Opisthorchiidae, Spirurida), as were lungworms since the flotation method and the SAFC technique are not the gold standard method for larvae detection for these parasites. Results of *Giardia duodenalis* testing (ELISA and PCR results combined) were also grouped according to age as described above (*n* = 13,892; Table 1). The result of *Cryptosporidium* spp. (ELISA and PCR results combined; *n* = 1,627) were divided into two age groups (< 1 year, ≥ 1 year) due to low numbers of positive samples (Table 1).

## Statistical methods

Statistical analyses were performed with PSPP (version GNU pspp 1.6.2). For comparison of infection rates and age groups, the Pearson's chi-square test was used (significance at *p* < 0.05). Fischer`s exact test was applied in cases when expected cell counts were < 5 in chi-square test. Yates` correction was used in one degree of freedom.

## Results

### Microscopical examination by flotation method and SAFC technique

Endoparasites were detected microscopically in 7.1% of the examined faecal samples. A monoinfection was found in 4.5%, two types of parasite stages were found in 2.1% and more than two types of parasite stages were found in 0.5% of the investigated samples. Of the detected parasite stages (*n* = 3,002) in the 2074 positive faecal samples, the majority were protozoa (49.3%) and nematodes (49.5%). Cestodes (1.1%) and trematodes (0.03%) were detected infrequently.

**Fig. 1:**
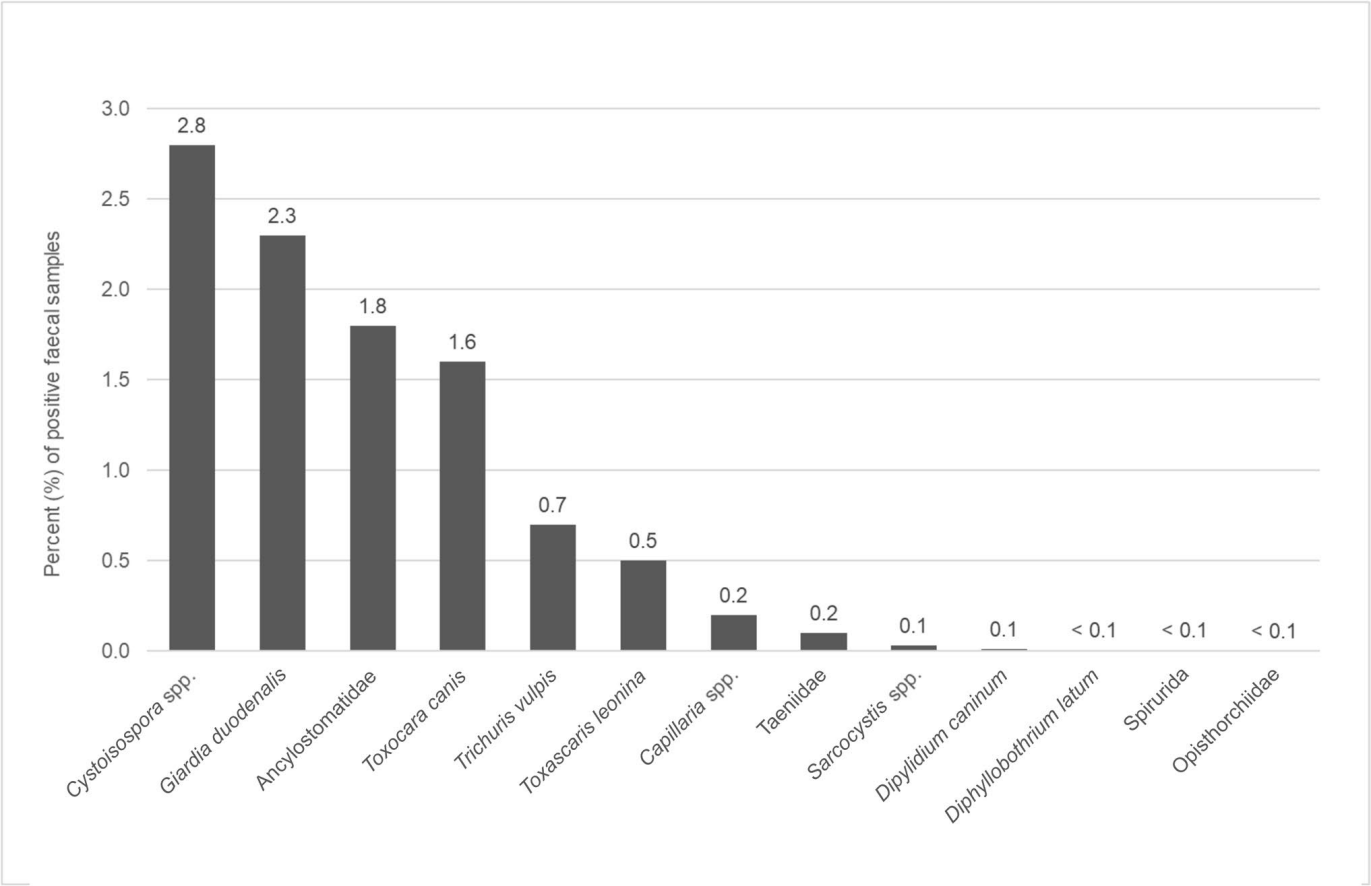
Detection rate of parasite stages in faecal samples of dogs (*n* = 29,219) detected by flotation method and SAFC technique

*Cystoisospora* spp. oocysts (2.8%) were found most frequently in the examined faecal samples, followed by *Giardia duodenalis* cysts (2.3%), Ancylostomatidae eggs (1.8%), *Toxocara canis* eggs (1.6%), *Trichuris vulpis* eggs (0.7%) and *Toxascaris leonina* eggs (0.5%). Other parasites, such as *Capillaria* spp. eggs (0.2%), *Angiostrongylus vasorum* larvae (0.2%), *Crenosoma vulpis* larvae (0.1%) and Taeniidae eggs (0.1%) were less frequently detected. Rare parasites included *Sarcocystis* spp. sporocysts (0.03%), *Dipylidium caninum* eggs (0.01%), *Diphyllobothrium latum* eggs (0.007%), Spirurida eggs (0.007%) and Opisthorchiidae eggs (0.003%) (Fig. 1). *Taenia* spp. and *Echinococcus* spp. eggs cannot be distinguished morphologically, so samples in which these eggs were identified were grouped together (family Taeniidae). Two of the samples with Taeniidae eggs could be further identified as *Echinococcus multilocularis* due to the presence of adults. Considerations regarding the detection of trematode and cestode eggs are dealt with in the discussion.

**Fig. 2:**
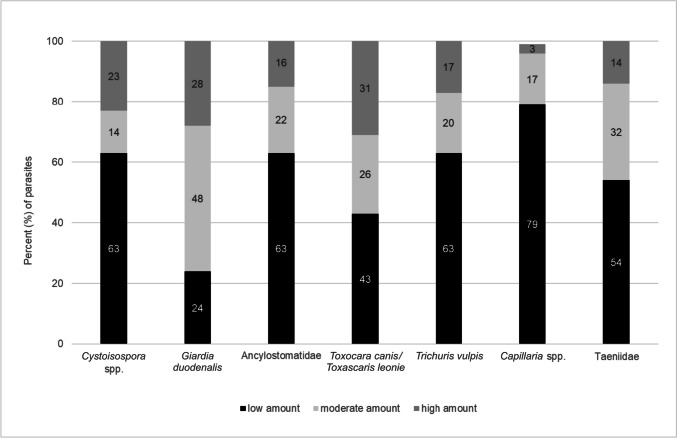
Amount of oocyst/cyst/egg excretion in faecal sample for the parasites detected: *Cystoisospora* spp. (*n* = 812), *Giardia duodenalis* (*n* = 660), Ancylostomatidae (*n* = 528), *Toxocara canis* and *Toxascaris leonina* (*n* = 612), *Trichuris vulpis* (*n* = 197), *Capillaria* spp. (*n* = 58) and Taeniidae (*n* = 28)

The excretion of oocysts, cysts and eggs was low in 50.8%, moderate in 26.0% and high in 23.3% of the total detected parasites (*n* = 3,002). Of the faecal samples in which *Cystoisospora* spp. were detected (*n* = 812), 63% had a low amount, 14% had a moderate amount and 23% had a high amount of oocysts. In samples with *Giardia duodenalis* (*n* = 660), 24% showed a low, 48% a moderate and 28% a high amount of cysts. Eggs were excreted in 63% in a low, 22% in a moderate and 16% in a high amount in samples with Ancylostomatidae (*n* = 528), in 43% in a low, 26% in a moderate and 17% in a high amount in samples with *Toxocara canis* and *Toxascaris leonina* (*n* = 472), in 63% in a low, 20% in a moderate and 17% in a high amount in samples with *Trichuris vulpis* (*n* = 197). A low amount of eggs were found in 79%, a moderate amount in 17% and a high amount in 3% of samples with *Capillaria* spp. (*n* = 58) and samples with Taeniidae eggs (*n* = 28) showed a low amount of excretion in 54%, a moderate amount in 32% and a high amount in 14% (Fig. 2).

### Microscopical examination for lungworm larvae by Baermann-Wetzel migration technique

Lungworm larvae were found in 1.1% of the samples via Baermann-Wetzel migration technique. These were identified as *Angiostrongylus vasorum* larvae in 0.75% of the samples and as *Crenosoma vulpis* in 0.3% of the samples.

### Detection of *Giardia duodenalis* and *Cryptosporidium* spp*. *coproantigen (ELISA) and DNA (PCR)

*Giardia* coproantigen was detected in 13.9% of the faecal samples and DNA was found in 19.4% of the examined faecal samples. *Cryptosporidium* coproantigen was detected in 1.0% of the samples and DNA was found in 2.0% of the examined samples.

### Comparison of different detection methods for *Giardia duodenalis*

In some samples, several tests were carried out to detect *Giardia duodenalis* (microscopic [flotation method and SAFC technique] and ELISA *n* = 8,121; microscopic and PCR *n* = 678; microscopic, ELISA and PCR *n* = 19; ELISA and PCR *n* = 11). In 19 samples examined microscopically, by ELISA and by PCR, all samples were negative for *Giardia duodenalis* cysts. Therefore, these 19 results of ELISA and PCR were presented together with the 11 samples for which only ELISA and PCR were performed. The results are given in Table 2.

**Table 2:** Comparison of microscopic examination (flotation method and SAFC technique), ELISA and PCR for the detection of *Giardia duodenalis* in faecal samples (microscopic and ELISA *n* = 8,121; microscopic and PCR *n* = 678; ELISA and PCR *n* = 30)

	ELISA		PCR	
	positive	negative	positive	negative
Microscopic				
positive	206 (2.5%)	17 (0.2%)	21 (3.1%)	1 (0.1%)
negative	623 (7.7%)	7,275 (89.6%)	77 (11.4%)	579 (85.4%)
ELISA				
positive	-	-	5 (16.7%)	1 (3.3%)
negative	-	-	3 (10.0%)	21 (70.0%)

### Age distribution

**Fig. 3:**
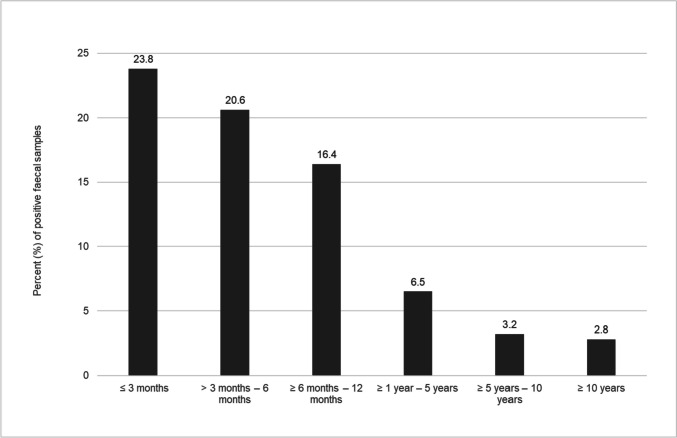
Detection rate of parasites in different age groups by microscopical examination with flotation method and SAFC technique (≤ 3 months: *n* = 1,134; > 3 months – 6 months: n = 1,489; ≥ 6 months – 12 months: *n* = 2,344; ≥ 1 year – 5 years: *n* = 10,759; ≥ 5 years – 10 years: *n* = 8,119 and ≥ 10 years: *n* = 5,374)

Based on the results of the microscopical examination via flotation method and SAFC technique, there was a significant difference in the rates of parasite detection when comparing the six age groups (*p* < 0.001). Dogs in the first year of life were more frequently infected with parasites (≤ 3 months: 23.8%, > 3 to 6 months: 20.6%, ≥ 6 to 12 months: 16.4%) compared to older animals (≥ 1 year to 5 years: 6.5%, ≥ 5 to 10 years: 3.2%, ≥ 10 years: 2.8%) (Table 3, Fig. 3). Detection rates in the first six months were significantly higher than in all other age groups (*p* < 0.001).

**Table 3:** Number of parasites detected per age group of 29,219 examined faecal samples (%)

Age group	No. of examined samples	*Cystoisospora* spp.	*Giardia duodenalis*	Ancylostomatidae	*Toxocara canis*	*Toxascaris leonina*	*Trichuris vulpis*	*Capillaria* spp.	No. of positive samples
≤ 3 months	1,134	167 (14.7)	90 (7.9)	25 (2.2)	62 (5.5)	6 (0.5)	4 (0.4)	0 (0)	270 (23.8)
> 3 – 6 months	1,489	108 (7.3)	136 (9.1)	58 (3.9)	94 (6.3)	27 (1.8)	12 (0.8)	5 (0.3)	307 (20.6)
≥ 6 – 12 months	2,344	106 (4.5)	171 (7.3)	87 (3.7)	111 (4.7)	53 (2.3)	46 (2.0)	14 (0.6)	385 (16.4)
≥ 1 – 5 years	10,759	237 (2.2)	191 (1.8)	237 (2.2)	123 (1.1)	41 (0.4)	96 (0.9)	25 (0.2)	702 (6.5)
≥ 5 – 10 years	8,119	126 (1.6)	40 (0.5)	78 (1.0)	52 (0.6)	13 (0.2)	24 (0.3)	11 (0.1)	262 (3.2)
≥ 10 years	5,374	68 (1.3)	32 (0.6)	43 (0.8)	30 (0.6)	0 (0)	15 (0.3)	3 (0.1)	148 (2.8)

The detection rate for each type of parasite (*Cystoisospora* spp., *Giardia duodenalis*, Ancylostomatidae, *Toxocara canis*, *Toxascaris leonina*, *Trichuris vulpis* and *Capillaria* spp.) individually also differed significantly (*p* < 0.001) between age groups, with significantly more frequent detection for each parasite in animals younger than 1 year (*p* < 0.001).

**Fig. 4:**
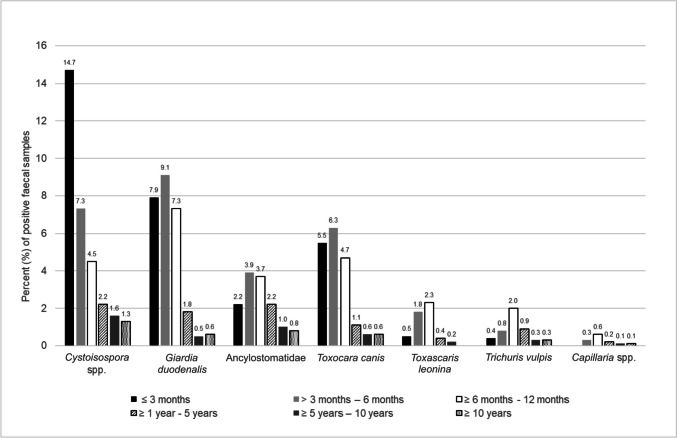
Detection rate of specific parasites (*Cystoisospora* spp., *Giardia duodenalis*, Ancyclostomatidae, *Toxocara canis*, *Toxascaris leonina*, *Trichuris vulpis*, *Capillaria* spp.) in different age groups by microscopical examination with flotation method and SAFC technique (≤ 3 months: *n* = 1,134; > 3 months – 6 months: *n* = 1,489; ≥ 6 months – 12 months: *n* = 2,344; ≥ 1 year – 5 years: *n* = 10,759; ≥ 5 years – 10 years: *n* = 8,119 and ≥ 10 years: *n* = 5,374)

*Cystoisospora* spp. were significantly (*p* < 0.001) more frequently found in dogs ≤ 3 months of age (14.7%) and the detection rate decreased continuously with age (Table 3, Fig. 4). Detection of *Giardia duodenalis* (≤ 3 months: 7.9%, > 3 to 6 months: 9.1%, ≥ 6 to 12 months: 7.3%) and *Toxocara canis* (≤ 3 months: 5.5%, > 3 to 6 months: 6.3%, ≥ 6 to 12 months: 4.7%) was high in the first year of life, with a steep decline in subsequent years. Within the first year of age, there was no significant difference between age group ≤ 3 months and age group > 3 to 6 months for *Giardia duodenalis* or *Toxocara canis* (*p* = 0.311 and *p* = 0.405, respectively). There was also no significant difference between age group ≥ 6 to 12 months and age group ≤ 3 months (*p* = 0.546 and *p* = 0.397, respectively), but a significant difference between age group ≥ 6 to 12 months and age group > 3 to 6 months (*p* = 0.047 and *p* = 0.041, respectively) for both parasites. The age groups > 3 to 6 months and ≥ 6 to 12 months had the significantly highest infection rates for Ancylostomatidae (3.9% and 3.7%, respectively; *p* = 0.019, *p* = 0.024) and *Toxascaris leonina* (1.8% and 2.3%, respectively; *p* = 0.006, *p* < 0.001) in animals younger than one year. The age group ≥ 6 to 12 months had significantly higher infection rates for *Trichuris vulpis* (2.0%) than the age group ≤ 3 months (*p* < 0.001) and the age group > 3 to 6 months (*p* = 0.006). Due to the low number of positive samples, the results for *Capillaria* spp., were combined into two age groups to test the differences between the groups. There was a significant difference (*p* < 0.001) between animals younger than one year (3.4%) and animals older than one year (1.5%).

**Fig. 5:**
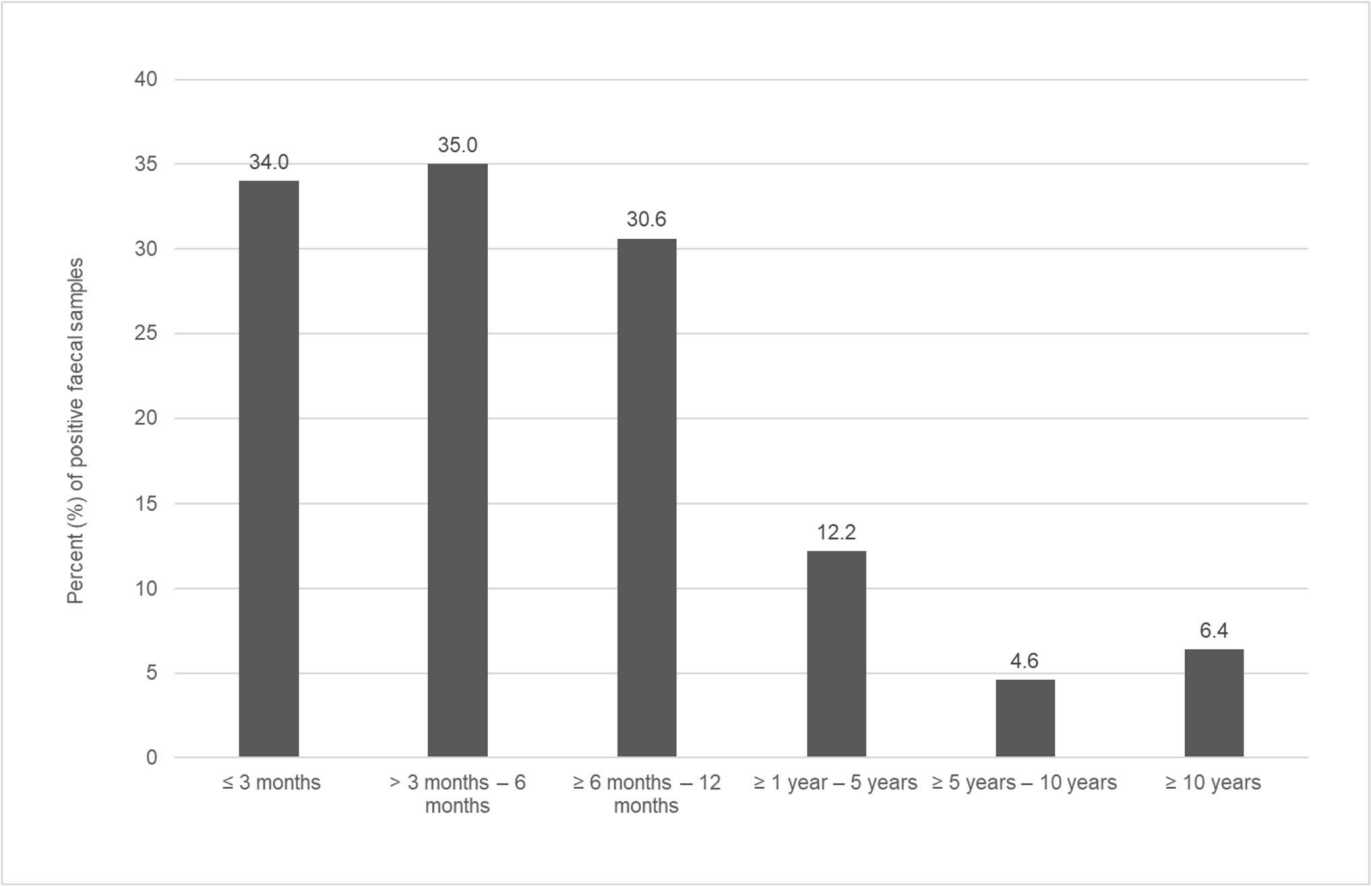
Detection of *Giardia duodenalis* in different age groups, results of ELISA and PCR combined (≤ 3 months: *n* = 692; > 3 months – 6 months: *n* = 1,109; ≥ 6 months – 12 months: *n* = 1,568; ≥ 1 year – 5 years: *n* = 5,144; ≥ 5 years – 10 years: *n* = 3,143 and ≥ 10 years: *n* = 2,236)

*Giardia duodenalis* detection by ELISA or PCR (Fig. 5), combining the results of both methods for the age groups, was also significantly (*p* < 0.001) higher in the first year of life (≤ 3 months: 34.0%, > 3 to 6 months: 35.0%, ≥ 6 to 12 months 30.6%). Within the first year, there was no significant difference between age group ≤ 3 months and > 3 to 6 months (*p* = 0.693) and age group ≤ 3 months and age group ≥ 6 to 12 months (*p* = 0.127), but the difference in detection rates between age group > 3 to 6 months and age group ≥ 6 to 12 months was significant (*p* = 0.019).

*Cryptosporidium* spp. (results of ELISA and PCR combined) was most frequently detected in the first year of life. However, the difference in detection rates in the group younger than one year (2.5%) and the group older than one year (1.0%) was not significant (*p* = 0.051), although in approached significance. However, the number of positive faecal samples was low (*n* = 21).

## Discussion

Retrospective analyses of large numbers of coproscopic examinations can provide overviews of endoparasite occurrence within a region, as well as how infection rate and types of parasites detected change over time compared with other studies. Nevertheless, comparisons of studies are difficult due to differences in examination methods and animal pools (age, housing conditions, healthy animals versus symptomatic animals).

Methods for detecting parasites vary in sensitivity. The sensitivity depends on the flotation medium, the eggs/gram of faeces and the amount of faeces used. With higher density flotation media (1.3 and 1.45) eggs of *Trichuris*, *Capillaria*, Taeniidae and Spirurida can be detected. The SAFC technique can be used for vegetative and cystic stages of protozoa such as *Giardia duodenalis*, as well as eggs and larvae of helminths. As this technique is also a sedimentation method, trematode eggs can be detected. However, the amount of faeces used is less than with sedimentation method or sedimentation-flotation method, which means that the sensitivity for detecting trematode eggs is lower (Deplazes et al. 2013). The irregular excretion of parasite eggs or the excretion of proglottids of cestodes (Martínez-Carrasco et al. 2007) also reduces the probability of detecting an infection. The detection rate of *Dipylidium caninum*, *Diphyllobothrium latum*, Spirurida and Opisthorchiidae is particularly affected by this detection problem in the present study. ELISA and PCR have a higher sensitivity for *Giardia duodenalis* detection than the flotation method, SAFC or MIFC technique (Cirak and Bauer 2004; Sommer et al. 2018; Symeonidou et al. 2020). In addition, faecal samples from an investigation pool containing a larger number of dogs under one year of age, contribute to a higher detection rate in the examined population, as puppies have a higher parasite load compared to adult dogs, especially with parasites such as *Cystoisospora* spp., *Toxocara canis* and *Giardia duodenalis* (Barutzki and Schaper 2003; Becker et al. 2012; Murnik et al. 2023).

In the present study, parasites were detected in 7.1% of 29,219 faecal samples using flotation method and SAFC technique. Similar results were reported in German laboratories (Elze et al. 2014; Raue et al. 2017), and in a prospective study of new arrivals of dogs in animal shelters (Becker et al. 2012). *Cystoisospora* spp. was the most frequently microscopically detected parasite in the present study, followed by *Giardia duodenalis*, Ancylostomatidae and *Toxocara canis*. Becker et al. (2012) and Raue et al. (2017) showed a higher detection rate for *Toxocara canis* and Raue et al. (2017) for *Cystoisospora* spp. as well. In contrast eggs of Ancylostomatidae were more often seen in the present study and the detection rate for *Giardia duodenalis* was higher as well, although Raue et al. (2017) and Becker et al. (2012) didn´t perform the SAFC technique in addition (Table 4).

**Table 4:** Detection rate of gastrointestinal endoparasites in dogs compared to other studies in Germany

	Samples of all age groups			Only dogs under 1 year	
	Present study	Raue et al. 2017	Becker et al. 2012	Present study	Murnik et al. 2023
Examined population	Retrospective, laboratory	Retrospective, laboratory	Prospective, stray and foster dogs	Retrospective, laboratory	Prospective
Methods used	Flot, SAFC	Combined Sed-Flot	Combined Sed-Flot	Flot, SAFC, *Giardia* ELISA and PCR*	Combined Sed-Flot, *Giardia* PCR, *Cryptosporidium* PCR
Analysed year	2019	2003–2012	2006–2007	2019	2020–2022
Examined sample number	29,219	2,731	445	4,967	386
Animals under 1 year	17%	N/A	26%	100%	100%
Parasite detection rates reported					
* Cystoisospora* spp.	2.8%	5.6%	2.5%	7.7%	7.3%
* Giardia duodenalis*	2.3%	0.6%	1.2%	14.9%	29.0%
Ancylostomatidae	1.8%	1.2%	0.9%	3.4%	0.3%
* Toxocara canis*	1.6%	2.8%	4.0%	5.4%	6.0%
* Toxascaris leonina*	0.5%	0.3%	0.2%	1.7%	0.5%
* Trichuris vulpis*	0.7%	0.7%	0.9%	1.2%	0.3%
* Capillaria* spp.	0.2%	0.4%	0.4%	0.4%	0.3%
Taeniidae	0.1%	0.4%	-	0.1%	0.8%
Positive samples	7.1%	11.0%	9.4%	25.1%	41.2%

*Abbreviations*: *Flot* flotation method, *SAFC* sodium acetate-acetic acid-formalin concentration technique, *combined Sed-Flot*  combined sedimentation-flotation technique, *N/A*  not available

*Of the 4,967 samples examined with flotation method and SAFC technique, in 35% (*n* = 1,750) samples *Giardia* ELISA or PCR was also performed

For better comparison, samples from dogs under one year of age were also analysed in the present study. Compared to the study by Murnik et al. (2023), eggs of Ancylostomatidae, *Toxascaris leonina* and *Trichuris vulpis* were detected more frequently (Table 4). In contrast, the detection rate for *Giardia duodenalis* was lower, but only 35% of the samples examined by flotation method and SAFC technique were additionally tested by *Giardia* ELISA or PCR.

The detection rate of lungworms was lower (1.1% vs. 3.5%) than previously published (Elze et al. 2014). However, Barutzki and Schaper (2003, 2011) showed similar results for lungworms in their analysis in 1999–2002 and 2003–2010 (1.2% and 0.9%, respectively) as the present study. A study conducted with dogs showing clinical signs suggestive of lungworm infection revealed a much higher infection rate of 13% (*Angiostrongylus vasorum* 7.4% and *Crenosoma vulpis* 6%) (Barutzki and Schaper 2009).

The occurrence of *Cryptosporidium* spp. was much lower than for *Giardia duodenalis*. The detection rate with ELISA was 1.0% and with PCR 2.0%. Raue et al. (2017) had 3.4% positive samples (examination requested by veterinarians), examined microscopically with carbol-fuchsin staining. In contrast, Cirak and Bauer (2004) had a high detection rate of 23% in animals from three animal shelters in Germany known to harbour endoparasites endemically. The dogs had no gastrointestinal signs. The authors mentioned the possibility of false positive results, as the microscopic examination with carbol-fuchsin staining revealed only 0.3% positive samples. Murnik et al. (2023) reported a much higher detection rate of 9.1% with PCR in dogs younger than one year, mainly from breeders and sampled in central Germany.

With regard to the age of the dogs examined, parasites were found significantly more frequently in animals in the first year of life compared to older animals, which corresponds to previous studies by Barutzki and Schaper (2003) and Becker et al. (2012). In another study by Barutzki and Schaper (2011), dogs under one year of age were divided into three age groups. They found similar results to the present study regarding which of the three age groups were significantly more frequently infected with the individual parasites in the first year of life. Murnik et al. (2023) divided the examined dog population under one year of age into four age groups. In contrast, they found no statistical association between positive faecal samples and different age groups in the first year of life, although the dog population studied was small (*n* = 387). Nevertheless, *Cystoisospora* spp. was mostly found in dogs aged ≤ 3 months, as in the present study and in the study by Barutzki and Schaper (2011). Other comparisons were not possible due to the different distribution of age groups in the study of Murnik et al. (2023).

Gates and Nolan (2009) reported an increase in the parasite prevalence in general in older dogs. For the individual parasites, this increase was only found for *Giardia duodenalis*. Possible explanations mentioned in their publication included the immune system with poorer immune response to parasites and the decreased use of chemotherapy in animals older than nine years. In the present study, an increase in parasite frequency in older dogs (between the age group ≥ 5 to 10 years and the age group ≥ 10 years) was significant for *Giardia duodenalis* detected by ELISA and PCR, but not for other parasites.

Although this study covers a large investigation pool of dogs throughout Germany and provides an overview of the frequency of parasites in diagnostic samples, some limitations must be considered. The results reflect the situation of a specific dog population as the samples are pre-selected: well-cared dogs, dogs with gastrointestinal diseases, faeces of dogs for parasite treatment controls and healthy dogs for routine examination. Another limitation is, that animals with a negative result are generally not re-tested to rule out a negative result during the prepatent period.

## Conclusion

The microscopic detection rate of protozoa and nematodes in the faeces of dogs examined in a diagnostic laboratory in Germany is similar to previous studies (Becker et al. 2012; Elze et al. 2014; Raue et al. 2017). *Cystoisospora* spp., *Toxocara canis* and Ancylostomatidae are the most common parasites found microscopically. Nevertheless, the use of ELISA and PCR resulted in the identification of *Giardia duodenalis* as the most common endoparasite in dogs. Dogs in their first year of life were more frequently infected with parasites than older animals. The highest detection rate was found in the first 6 months of life. Parasites of a potentially zoonotic nature, such as *Toxocara canis* and *Giardia duodenalis*, are more common in young animals. Breeders and owners of puppies should therefore be aware of the risk of infection, even if the risk is low. It is important to continue owner education and parasite control.

## Data Availability

The raw data supporting the conclusions of this article will be made available by the authors, without undue reservation.
